# Dimensionality Reduction in Complex Medical Data: Improved Self-Adaptive Niche Genetic Algorithm

**DOI:** 10.1155/2015/794586

**Published:** 2015-11-16

**Authors:** Min Zhu, Jing Xia, Molei Yan, Guolong Cai, Jing Yan, Gangmin Ning

**Affiliations:** ^1^Department of Biomedical Engineering, Zhejiang University, 38 Zheda Road, Hangzhou, Zhejiang 310027, China; ^2^Guizhou Key Laboratory of Agricultural Bioengineering, Guizhou University, Guiyang, Guizhou 550025, China; ^3^Zhejiang Hospital, Hangzhou, Zhejiang 310058, China

## Abstract

With the development of medical technology, more and more parameters are produced to describe the human physiological condition, forming high-dimensional clinical datasets. In clinical analysis, data are commonly utilized to establish mathematical models and carry out classification. High-dimensional clinical data will increase the complexity of classification, which is often utilized in the models, and thus reduce efficiency. The Niche Genetic Algorithm (NGA) is an excellent algorithm for dimensionality reduction. However, in the conventional NGA, the niche distance parameter is set in advance, which prevents it from adjusting to the environment. In this paper, an Improved Niche Genetic Algorithm (INGA) is introduced. It employs a self-adaptive niche-culling operation in the construction of the niche environment to improve the population diversity and prevent local optimal solutions. The INGA was verified in a stratification model for sepsis patients. The results show that, by applying INGA, the feature dimensionality of datasets was reduced from 77 to 10 and that the model achieved an accuracy of 92% in predicting 28-day death in sepsis patients, which is significantly higher than other methods.

## 1. Introduction

Clinical decision system is able to aid in diseases diagnosis and predict the clinical outcomes in response to treatment [1, 2]. For the diagnosis of sepsis, a number of scoring systems have been proposed, such as the Acute Physiology and Chronic Health Evaluation (APACHE), Sequential Organ Failure Assessment (SOFA), and Clinical Pulmonary Infection Score (CPIS) [1, 3]. They are challenged because traditional markers of infection mislead and there is lack of better evaluation methods for prognosis [1, 4–6]. To improve the outcome of treatments, diagnostic models are needed to accurately predict the development of sepsis as well as stratify its severity [7].

However, the clinical data of sepsis involved in diagnostic models are usually high dimensional. High-dimensional datasets increase the complexity of classification and reduce the effect of models [8]. Thus, before building models, it is necessary to reduce the data dimension while retaining essential information of the original data. Feature extraction and feature selection are the main methods in dimensionality reduction [2, 9].

*(A) Feature Extraction*. Feature extraction transforms the original feature space into a new one of lower dimension. Algorithms like Principal Component Analysis (PCA), Multidimensional Scaling (MDS), and Independent Component Analysis (ICA) are widely used for feature extraction. However, ICA and PCA are linear projection methods, and if the feature vectors distribute along a nonlinear manifold in a high-dimensional space, they might lead to classification errors [10, 11]. Besides, MDS is sensitive to undersampling datasets and has difficulty in dealing with defect data [12]. Furthermore, PCA, MDS, and ICA will generate new parameters after dimensionality reduction, and the significance of the new parameters is not always interpretable.

*(B) Feature Selection*. Feature selection is a kind of process that selects an optimal feature subset from the original features, which retains sufficient information [13]. Currently, quite a lot of feature selection algorithms have been developed, such as Genetic Algorithms (GAs), Support Vector Machines (SVM) Wrapper, Sparse Generalized Partial Least Squares Selection (PLS), and Particle Swarm Optimization (PSO) [14–17]. Among them, GAs are popularly utilized. However, in some multimodal optimization problems, GAs failed to maintain multiple global or local optima [13]. Thus many efforts have been made to improve the ability of GAs in achieving multiple peak solutions, by adding scaling fitness and adjusting fitness competence rule [18].

*(a) GAs*. Genetic Algorithms have been used to reduce the numbers of features in datasets [19–21]. Genetic Algorithm Pipe Network Optimization Model (GENOME) has been applied to optimize the design of new looped irrigation water distribution networks [22]. An online web-based feature selection tool (DWFS) was developed according to the GA-based wrapper paradigm [23]. However, when using GAs [24–26], it is difficult to handle problems such as nonlinear, singular, and multimodal ones. The key issue is that the population is easily trapped in a limited number of solutions; and premature solutions have no capability to obtain better results [18]. Therefore, the Niche Genetic Algorithms (NGAs) are introduced to build a better environment to resolve the problem.

*(b) NGAs*. The capability to locate multiple loci often permits NGAs to be robust and effective in solving multimodal optimization problems [27–29]. The Twin-space Crowding Genetic Algorithm (TCGA) and Game-Theoretic Genetic Algorithm (GTGA) are introduced in the literature [18, 30]. The reported work [31] showed that the Nondominated Sorting Genetic Algorithm (NSGA) lacks elitism and needs to specify the sharing parameter [32]. However, most niche methods require prior knowledge such as the niche radius or the distance threshold. Accordingly, the niche distance is either set randomly or set as fixed value in advance. These technologies are unable to adaptively obtain the niche distance following evolution and prone to eliminate the potentially excellent individuals [33, 34].

To address the problems, we proposed Improved NGA (INGA) algorithm with embedded self-adaptive niche-culling mechanism for dimensionality reduction. Since MDS and PCA are the typical feature extraction algorithms while GA and NGA are the typical feature selection algorithms, we compared the dimension reduction results of them with INGA to verify the validity of INGA in dimension reduction. By applying INGA, the improvement in the accuracy rate of sepsis diseases classification is noteworthy, while the data dimension is reasonably reduced.

## 2. Method

The idea of NGA is applying the biological concept of a niche to evolutionary computations. It shows a survival environment with a prespecified distance parameter *L*. The *L* of NGA is set in advance, only allowing a single excellent individual in this distance. NGA has the following main disadvantages.

(1) A fixed distance parameter affects the convergence rate. If the value of *L* is too large, there will be lots of individuals within this distance and they need to be culled. This will lower the convergence rate. In contrast, if the value of *L* is too small, there are no sufficient individuals and this will lead to premature convergence.
(2) Single individual will inhibit potential individuals. Within the distance *L*, only one single excellent individual is allowed and it will cause the elimination of potentially excellent individuals and make the result of the dimension reduction too large.
(3) The diversity of the subpopulations is insufficient. Population diversity is closely related to subpopulations scale, but the subpopulations scale of NGA is set in advance and cannot be adjusted. It is difficult to find an optimum scale of subpopulations. As a result, if the subpopulations scale is too large, the diversity of the population is easy to be destroyed; on the contrary, the additional calculation of the algorithm will be increased.

To address these problems, we developed Niche Elimination Operation, as shown in the part (A). Afterwards, INGA is constructed, as shown in part (B) (Figure 2).

*(A) Niche Elimination Operation*

*(a) Self-Adaptive Survival Distance*. The distance parameter *L* is designed to be self-adaptive with the Euclidean distance among individuals of each generation to avoid the convergence problem caused by preset *L*:

(1) D=Xi−Xj=∑k=1lenxik−xjk2,i,j∈1,2,…,M,  i≠j.

*X*_*i*_ and *X*_*j*_ are two individuals of the current population, which are made up of loci genetics. *M* is the number of individuals in the current population. len is the number of loci, which is used to form and evaluate the lengths of individuals. *x*_*ik*_ and *x*_*jk*_ are the values of loci. The distance parameter *L* is calculated by

(2) $$\begin{array}{c} L=\min\left\{\;D\;\right\}. \end{array}$$

Because individuals of each generation are different and the values of the distance parameter vary with generation, a reasonable distance parameter will be obtained in the evolutionary process of each generation to get a better niche environment.

*(b) Similarity Criterion*. Allowing one single excellent individual within *L*, this will cause the elimination of potentially excellent individuals which may not be similar to the retained excellent. So, within the distance parameter *L*, the similarities of biallelic loci are used to judge the similarity of the individuals and determine whether the individuals should be retained.

The similarity of biallelic loci and average similarity between two individuals are given by the following two equations:

(3) SDXi,Xj=∑k=1lennumXik==Xjklen,i∈1,2,…,M,  j∈i+1,i+2,…,M,

where SD(*X*_*i*_, *X*_*j*_) represents the similarity between two individuals, *X*_*i*_ and *X*_*j*_. num(*X*_*ik*_ =   = *X*_*jk*_) is the number of the same allele value of two individuals. Consider

(4) MSDi=∑j=i+1MSDXi,Xjlen∗M−1,i∈1,2,…,M,  j∈i+1,i+2,…,M.

MSD_*i*_ represents the average similarity between the *i*th individual and the others. When ‖*X*_*i*_ − *X*_*j*_‖ < *L*, the similarity between two individuals will be distinguished. If the similarity is larger than the average similarity, the individual that has a lower fitness will be given a penalty function, as shown in the following equation. Otherwise, the lower fitness individuals can be retained:

(5) $$\begin{array}{c} f_{j}^{\prime }\left(\;X\;\right)=f_{j}\left(\;X\;\right)*P, \end{array}$$

where *f*_*j*_(*X*) is the original fitness of the individual, *f*_*j*_′(*X*) is the new fitness, and *P* is the penalty function (usually 10^−30^). This method can reduce the elimination of individuals.

*(c) Maintain Population Diversity*. To maintain the diversity of the population, the scale of the subpopulations should be controlled. So (6) and (7) are designed with a memory pool of optimal individuals to limit the scale for the subpopulations of each generation:

(6) $$\begin{array}{c} f\left(\;t\;\right)=\overset{M_{\left(t\right)}}{\underset{i=1}{\sum }}\frac{f_{i}\left(t\right)}{M_{\left(t\right)}}, \end{array}$$

where *f*(*t*) represents the average fitness value of generation *t*, *f*_*i*_(*t*) represents the fitness of individual *i* in generation *t*, and *M*_(*t*)_ is the scale of the population in generation *t*. Thus, the scale of subpopulations in generation *t* + 1 is *M*_(*t*+1)_. This is calculated as

(7) $$\begin{array}{c} M_{\left(t+1\right)}=M_{\left(t\right)}\cdot f\left(\;t\;\right)\cdot \frac{t}{\sum_{i=1}^{t}f\left(i\right)}. \end{array}$$

A memory pool of optimal individuals is designed to exchange excellent evolutionary individuals. The operation increases the possibility of obtaining more excellent individuals, and to some extent, avoids the problem of premature convergence during the evolutionary process of a single population. The individuals of general *t* + 1 are sorted by fitness, and the formers *N* are put into the memory pool.

Through the result of *M*_(*t*+1)_, the ability of maintaining the population diversity, *d*(*p*), is designed as in the following two equations. The smaller the value of *d*(*P*) is, the higher its population diversity is:

(8) $$\begin{array}{c} d\left(\;p\;\right)=\frac{\sum_{1}^{t}{d\left(P\right)}_{t}}{t}, \end{array}$$

where *d*(*P*)_*t*_ is the capability to maintain the population diversity in generation *t*. And *d*(*P*)_*t*_ is designed as follows:

(9) $$\begin{array}{c} {d\left(P\right)}_{t}=\frac{1}{l\cdot M_{\left(t\right)}}\overset{l}{\underset{j=1}{\sum }}\max\left\{\;\overset{M_{\left(t\right)}}{\underset{i=1}{\sum }}\left(\;1-a_{ij}\;\right),\;\;\overset{n}{\underset{i=1}{\sum }}a_{ij}\;\right\}, \end{array}$$

where *l* is the length of the individual encoding, *M*_(*t*)_ is the scale of the population in generation *t*, and *a*_*ij*_ is the *j*th loci of the *i*th individual.

*(B) Flowchart of INGA*

**Step 1 (calculate fitness).**

At first, *M* initial individuals are produced at random. Usually, it takes the reciprocal of the sum of error square of the classifier test set data as fitness function [33] in order to fully reflect the advantage of controlling errors by combining INGA with classifier:

(10) $$\begin{array}{c} f\left(\;X\;\right)=\frac{1}{\sum_{i=1}^{n}{\left({\hat{t}}_{i}-t_{i}\right)}^{2}}, \end{array}$$

where $\hat{t}$ is the predicted value of test set, *t* is the true value of test set, and *n* is the sample number of test set. Individuals are sorted by fitness in descending order, and the former *N* individuals are remembered in the memory pool (*N* < *M*).

**Step 2 (Niche Elimination Operation to produce excellent initial individuals).**

**Figure 1:**
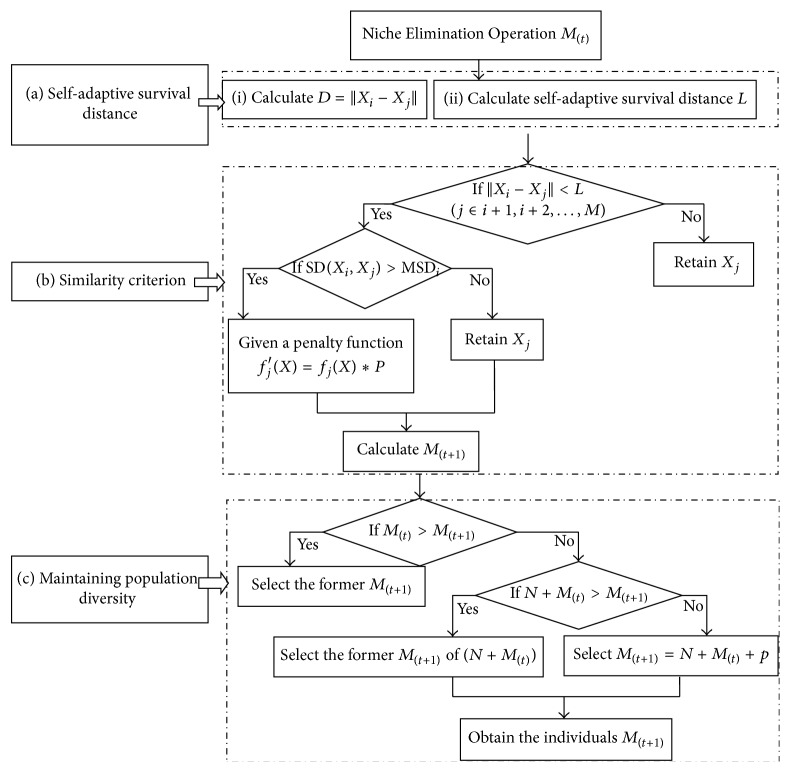
Niche Elimination Operation.

**Figure 2:**
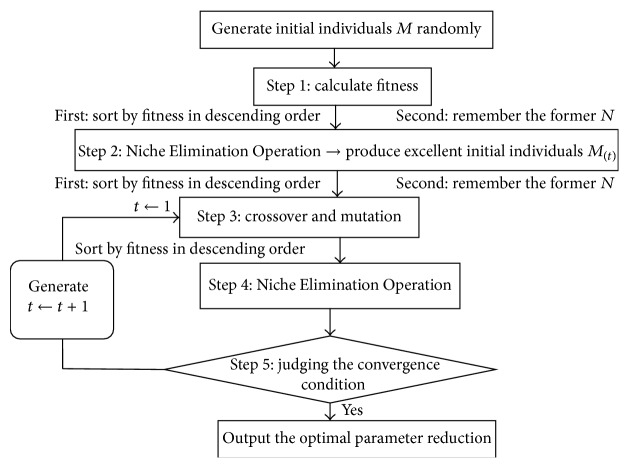
Flowchart of INGA.

In this step, the excellent initial individuals *M*_(*t*)_ are produced, as shown in Figure 1.

(a) *Self-Adaptive Survival*. First, calculate the Euclidean distance *D* between *X*_*i*_ and *X*_*j*_ according to (1). Second, calculate self-adaptive survival distance *L* according to (2).
(b) *Similarity Criterion*. Judge the similarity of the individuals within the distance *L* according to the method of allele contrast, so as to determine whether the individual should be retained. When ‖*X*_*i*_ − *X*_*j*_‖ < *L*, the similarity of biallelic loci and average similarity between two individuals are compared. If they are not similar, the individual of lower fitness needs not to be eliminated. The similarity of biallelic loci SD(*X*_*i*_, *X*_*j*_) and average similarity MSD_*i*_ between two individuals are given by (3) and (4). When SD(*X*_*i*_, *X*_*j*_) > MSD_*i*_, then *f*_*j*_(*X*) is punished, using a penalty function *f*_*j*_′(*X*) = *f*_*j*_(*X*)*∗P* according to (5). If not, the individual with lower fitness will be retained. On the other hand, when ‖*X*_*i*_ − *X*_*j*_‖ > *L*, the individual with lower fitness will be retained.
(c) *Maintaining Population Diversity*. According to (7), the number of subpopulations *M*_(*t*+1)_ is calculated. Individuals are sorted by fitness in descending order, if the scale of the existing subpopulation *M*(*t*) is larger than *M*(*t* + 1), select the individuals *M*_(*t*+1)_; otherwise, *N* individuals are merged in the memory pool with the existing subpopulations and sorted by fitness in descending order; when *N* + *M*_(*t*)_ > *M*_(*t*+1)_, the former individuals *M*_(*t*+1)_ of (*N* + *M*_(*t*)_) are selected; when *N* + *M*_(*t*)_ < *M*_(*t*+1)_, *P* individuals will be generated randomly; individuals *M*_(*t*+1)_ are selected, on the condition that *M*_(*t*+1)_ = *N* + *M*_(*t*)_ + *p*. Through this method, the initial population will have a higher average fitness and will be conducive to the evolution of population towards the solution of the problem.

**Step 3 (self-adaptive crossover and mutation operation).**

Considering the probability of crossover and mutation, it is too small to escape from making the system fall into the local optimal solution, and if it is too large, it can escape from the local optimal solution but is prone to instability and convergence because the count of crossover and mutation is so frequent. In order to improve this shortcoming, the equations of self-adaptive crossover (*P*_*c*_) and mutation probability (*P*_*m*_) are used [35, 36]:

(11) Pc=Pc1−Pc1−Pc2fmax−favgf′−favgf′≥favgPc1f′<favg,

(12) Pm=Pm1−Pm1−Pm2fmax−favgf−favgf≥favgPm1f<favg.

*f*_max_ is the maximum fitness value; *f*_avg_ is the average fitness value of each population; *f*′ is the larger fitness value of the two individuals crossing; and *f* is the fitness value of individuals of mutation. *P*_*c*1_, *P*_*c*2_ are, respectively, the crossover probability value of two individuals; *P*_*m*1_ and *P*_*m*2_ are the mutation probability values of two individuals.

**Step 4 (Niche Elimination Operation).**

After the self-adaptive crossover and mutation operation, put the new individual into the Niche Elimination Operation again to obtain the optimal individual, as shown in Figure 1.

**Step 5 (judging the termination condition).**

If it does not meet the termination condition, then update the counter *t* as *t* + 1 and make the population in Step 4 be the new next generation population, and then go to Step 2. If the termination condition is satisfied, output the optimal dimensionality reduction parameters selected.

## 3. Dataset Description

Experiments are conducted on a sepsis dataset, for which data are gathered from Zhejiang Hospital. The goal of the classifier was to determine, based upon the test results provided, whether a patient should be diagnosed as 28-day death [37]. The number of samples in the two classes was balanced. The training set contained 124 negative (28-day death) cases and 173 positive cases. Likewise, the testing set consisted of 77 negative samples and 123 positive ones. Data are organized in a table with 77 columns for attributes of patients and 497 rows for specific samples. There are missing values in this table because some questions have not been answered, so we replaced them with 0. There is not any correlation among attributes, and this creates an orthogonal space for using Euclidean distance. All samples include the same number of attributes [13].

## 4. Experimental Setup

This work used the PCA, MDS, NGA, and INGA to reduce the dimensionality of the dataset, and the selected algorithms were also combined with three classic classifiers, Random Forest (RF), Support Vector Machine (SVM), and Back Propagation (BP). The experimental setup is as follows.

*Set the Initial Population Scale*. The literature [37, 38] suggests that an optimal initial population should number from 20 to 100; the present work takes 90 as the initial population *M*, considering the computation time and the range of the search. The stored individuals *N* in Niche Genetic Algorithm are usually selected as one-thirds of population scale. The probability of crossover is determined by (11), and the mutation probability is determined by (12).

**Figure 3:**
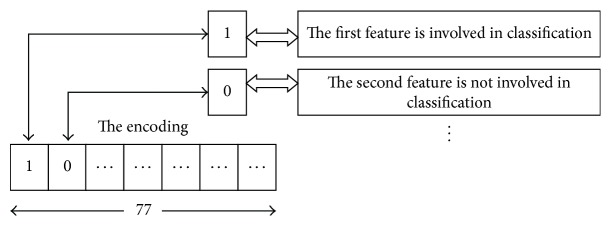
The relationship between encoding and features.

*Set the Encoding*. The data are organized in a table with 77 columns for attributes of patients and each bit is assigned to one feature; thus the encoding length is designed as 77. If the *i*th bit equals 1, then the *i*th feature is involved in classification; otherwise, the corresponding feature is not involved, as shown in Figure 3.

*Set the Convergence Condition*. The evolutional generation is set to 100 according to the previously published works [13, 37]. The fitness function is the reciprocal of the sum of the prediction error square of the model. Convergence is achieved when the largest and least fitness values are equivalent. This paper adopts the maximum evolutional generation and convergence degree of the population to construct the condition of algorithm convergence: end the calculation when it can meet one of the two conditions; namely, the evolutional generation reaches the preset values or population convergence appears [36].

*Set the Experiment Running Time*. The experiment used *k*-fold cross-validation, 80% of the samples were randomly selected as the training set, and the rest were used as the test set. The experiment was repeated 100 times [39].

## 5. Result

The clinical manifestation of the sepsis disease is complicated, and it is difficult to accurately determine the 28-day mortality. This study applies the improved self-adaptive Niche Genetic Algorithm to the diagnosis process of septic 28-day mortality, using dimensionality reduction to obtain the optimal feature parameters and improve the diagnostic precision. Here, premature state, population distribution, accuracy of classification, and robustness have been used to measure the quality of the algorithms.

*(A) Premature State*. Avoiding premature state is a standard of the algorithms; premature means that the performance is as follows: (a) the population diversity is reduced, (b) the convergence ability is low, and (c) the convergence rate is low. Thus, we used these factors to measure whether the algorithms were premature or not.

*(a) Population Diversity*. The Schaffer function, presented as (13), is used to generate data, and the results of population diversity, with *d*(*P*) calculated with (9), are shown in Table 1:

(13) fx1,x2=0.5−sin2⁡x12+x22−0.51+0.001x12+x222,−100≤xi≤100  i=1,2.

We can see from Table 1 that the value of *d*(*P*) of INGA is smaller than that of GA and NGA under the condition of the same evolution generations, demonstrating the advantages of INGA in maintaining the population diversity.

*(b) Convergence Ability*. Convergence ability means the ability to obtain global optimal values when algorithm stops. We know from the properties of the Schaffer function that the global maximum is 1 and that two local maxima near the maximum value are 0.99028 and 0.96278. If the maximum value was larger than 0.999, we can judge the convergence appearance, and the global solution is obtained. When local maxima values are obtained, we can judge that there is no convergence, as only the local solution is obtained. Thus, GA, NGA, and INGA are used to obtain the maximum value of the Schaffer function, as shown in Table 2.

From the data in Table 2, we can see that, in the 10 independent experiments, it is easier for GA and NGA to fall into two local maxima. There are 10 times for INGA to search the global optimal value, there are 7 times for NGA to search the global optimal solution, and GA only has 4 times, which means that there is a certain gap between the ability of these two algorithms to search for the global optimal solution compared with INGA.

**Figure 4:**
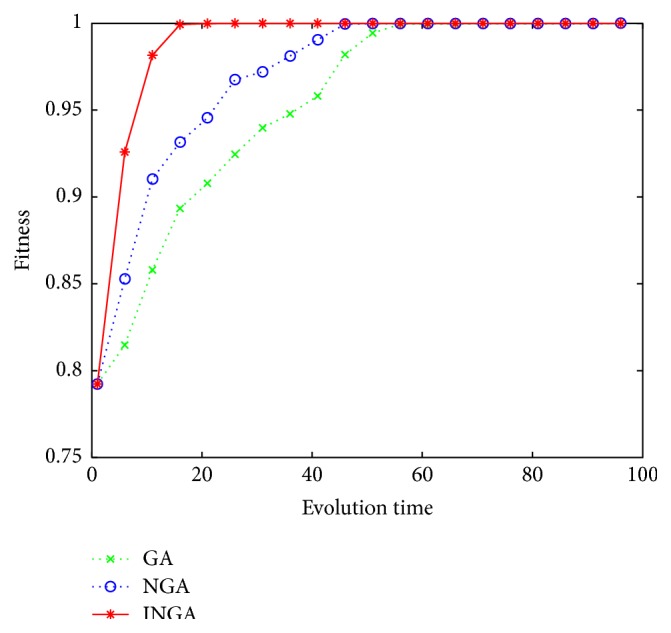
Convergence curves.

*(c) Convergence Rate*. The comparison of convergence curves among GA, NGA, and INGA is shown in Figure 4. We can see from Figure 4 that INGA has the fastest convergence rate. It has converged to the average fitness by the 20th generation. The remaining two algorithms converged to the average fitness by the 42th and 67th generations, respectively.

*(B) Population Distribution*. In Section 2, self-adaptive survival distance is used to set up the distance of NGA, and criterion similarity is used to determine whether the individual is retained or not. Both of them constitute the population distribution. So the figure of population distribution is built to assess the effect of the self-adaptive survival distance and criterion similarity methods.

**Figure 5:**
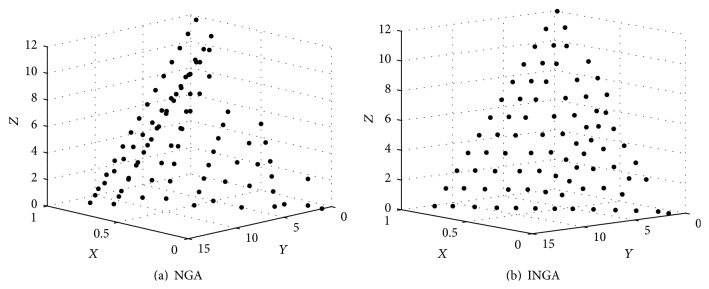
Individuals' distribution, where the *x*-axis represents the fitness of the individuals and the *y*- and *z*-axes represent the Euclidean distance between the individuals.

Figure 5 is the population distribution within the niche distance. It shows that the final population obtained by INGA can be more uniformly distributed; thus self-adaptive survival distance and similarity criterion designed in this paper is adaptive.

*(C) Dimensionality Reduction and Classification*. To assess the performance of dimensionality reduction, PCA, MDS, NGA, and INGA were embedded in the BP, SVM, and RF classifiers to carry out the classification diagnosis. The accuracy of classification and ROC curve diagram are shown as follows.

**Figure 6:**
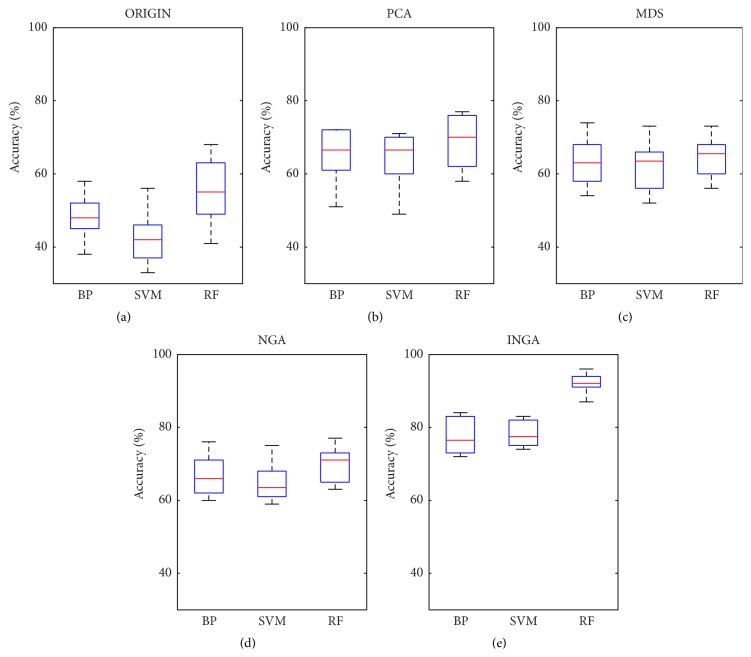
Classification accuracy (%). (a) is the result before dimensionality reduction and (b)–(e) are the result after dimensionality reduction.

*(a) Accuracy of Classification*. The number of feature subsets before and after dimensionality reduction is shown in Table 3. It is shown that INGA has better control over the number of feature subsets than other dimensionality reduction methods, as a smaller number of feature subsets were obtained by INGA. However, considering the number of feature subsets alone is not enough, as the classification accuracy should be combined. The classification accuracies before and after dimensionality reduction are shown in Figure 6. It is noticed that the accuracy increased obviously after the dimensionality reduction; the highest accuracy was obtained by RF-INGA.

**Figure 7:**
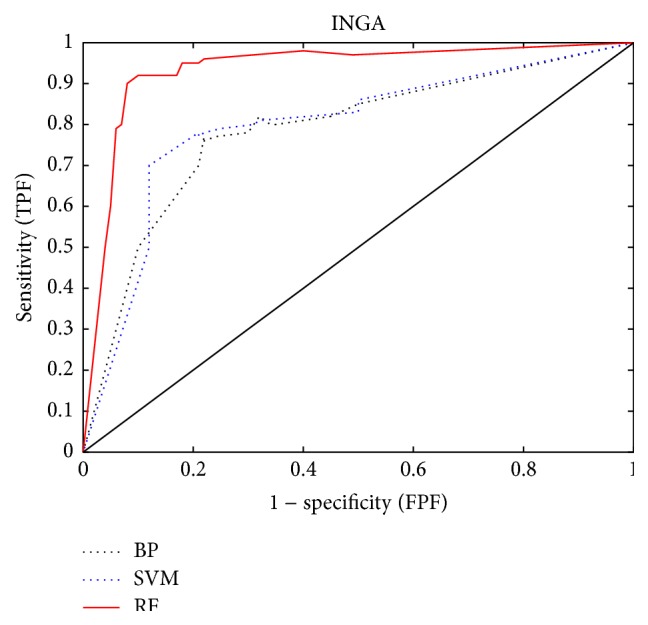
ROC curves. Classification using BP, SVM, and RF based on the INGA dimension reduction algorithm.

**Figure 8:**
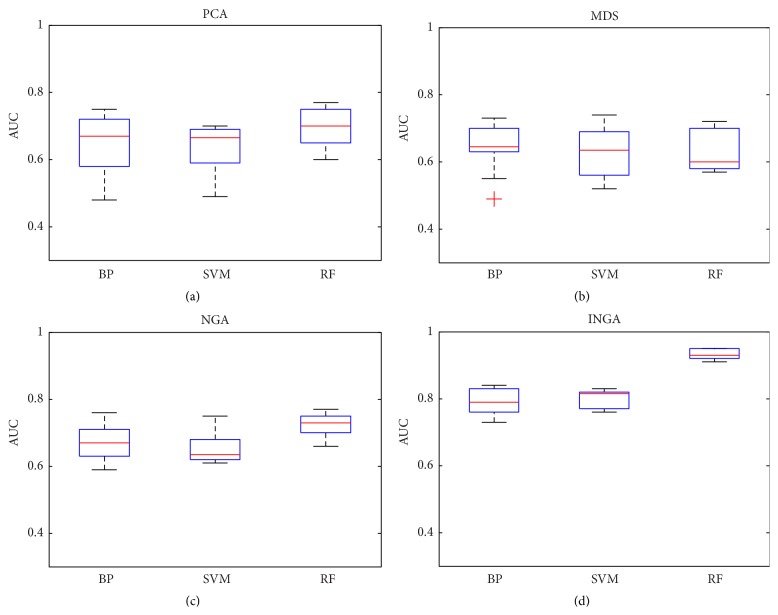
Area under the curve (AUC) of the algorithm.

*(b) The ROC Curves*. The receiver operating characteristic (ROC) curve and area under the curve (AUC) are shown in Figures 7 and 8.

From Figures 7 and 8, we can see that INGA yields a better result and that the covered areas of ROC offer an obvious improvement compared with PCA, MDS, and NGA. At the same time, the highest AUC was obtained by the RF classifier after INGA dimensionality reduction.

*(D) Robustness*. The robustness of the algorithm was tested by introducing random noise in the data. The* k*-fold cross-validation method was used to compare the effects of noise. 5% of the samples, selected randomly from the training set, and their labels are changed, used as noise samples. The operation was repeated 100 times, and the average value was taken to compare the classification accuracy.

**Figure 9:**
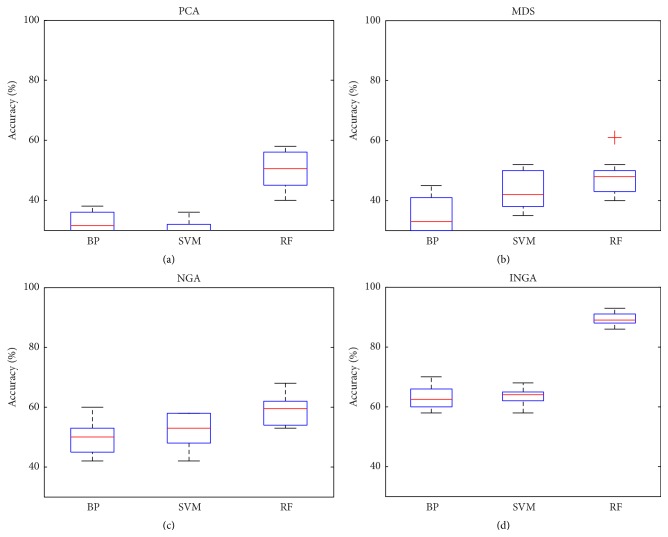
Robustness (%) of the algorithm.

From Figure 9, we can see that noise poses a significant effect on the dimensionality reduction methods of PCA and MDS. In comparison with Figure 6, the accuracy of the three classifiers decreased by 18% to 35%; on the contrary, INGA is less affected by the noisy conditions, and the accuracy of the three classifiers with INGA only decreased by 3% to 13%. The robustness of the INGA algorithm is strengthened, and its antinoise ability is the best, especially when it is combined with RF.

## 6. Discussion

The integrated feature selection algorithms and classification accuracy were valid on clinical sepsis data. INGA exhibited advantages in feature selection over other approaches, and, moreover, INGA-RF obtained classification accuracy higher than 90% in identifying the death of sepsis patients, showing the best performance of all of the techniques and using only 10 features.

The present work has proposed an improved INGA algorithm to resolve the premature state in traditional GA and NGA, which are characterized as having reduced population diversity, weak convergence ability, and low convergence rate. As shown in Table 1, regarding *d*(*P*), a measure of population diversity, INGA has the smallest value of 0.5629 as compared with GA and NGA. As shown in Table 2, for 10 independent experiments, INGA achieved the global optimum in all experiments, while NGA and GA succeeded in only 7 and 4 experiments, respectively. Figure 4 shows the convergence rate estimated by the generations of convergence. INGA had the fastest convergence rate with 20 generations, while 42 and 67 generations were required for GA and NGA, respectively. These findings suggest that INGA is superior overall to the other methods.

The dominating performance of INGA in avoiding premature convergence is owed to the following improvements in the work: (i) the introduction of the self-adaptive survival distance: differing from the conventional methods, the survival distance is automatically adjusted in the evolutionary process of each generation; this ensures reasonable distance parameters and leads to an adaptive niche environment; this approach can obtain more reasonable individuals with excellent global optimization ability and high convergence speed; (ii) the application of a similarity criterion that retains more reasonable individuals: the similarity of biallelic loci was used to decide whether the individuals in the neighborhood should be retained; this approach can harvest more reasonable individuals, increasing the possibility of finding the global optimal solution; and (iii) the use of a memory pool for optimal individuals: a pool was designed to reserve and exchange excellent evolutionary individuals for each generation; this maintains the diversity of the population and increases the quantity of excellent individuals; to some extent, it also avoids the problem of premature convergence during the evolutionary process of a single population.

The testing results on clinical sepsis cases show that, combined with INGA, three types of classifiers achieved the accuracies in predicting 28-day death of 92% (RF), 78% (SVM), and 77% (BP), respectively. In contrast, the highest accuracy of the classifiers employing NGA, PCA, and MDS is only 70%. This suggests that INGA is effective in improving the performance of classifiers for complex clinical datasets.

However, it is worth pointing out that the present work has some limitations. First, the validity of INGA was only tested in sepsis patients. Although the algorithm is generally functional, it is necessary to investigate the effectiveness of INGA on further datasets. Second, the coherence between INGA and the classifiers remains unclear. Our work revealed that the RF method with embedded INGA is mostly satisfied. One question that may arise is how to figure out the optimum combination of the dimension reduction algorithm and classifier. This question is out of the scope of the current work, which is focused on the dimension reduction. However, it should be clarified in a further study.

## 7. Conclusion

This paper proposed an improved algorithm for feature reduction in high-dimensional data. The methods were imbedded in classifiers to predict the prognosis of sepsis patients based on complex clinical datasets. The results indicate that the improved NGA, INGA, is most effective in reducing the number of attributes and enhancing the convergence speed compared to other commonly used algorithms, such as PCA, MDS, and NGA. Moreover, INGA associated with RF to achieve the highest accuracy in assessing the severity of sepsis. This suggests that INGA has the potential for complex data processing, particularly for medical pattern recognition.

## Figures and Tables

**Table 1:** *d*(*P*): the ability to maintain population diversity. *d*(*P*) was run for 20, 50, and 100 generations, respectively, in GA, NGA, and INGA.

Generation	GA	NGA	INGA
20	0.5635	0.5213	0.4812
50	0.6271	0.5748	0.5248
100	0.6963	0.6147	0.5629

**Table 2:** Convergence of the Schaffer function.

Execution count	GA		NGA		INGA	
	Optimal value	Whether converges	Optimal value	Whether converges	Optimal value	Whether converges
1	0.9903	N	0.9903	N	0.9995	Y
2	0.9632	N	0.9998	Y	1	Y
3	1	Y	1	Y	0.9998	Y
4	0.9619	N	0.9625	N	1	Y
5	0.9991	Y	0.9991	Y	0.9995	Y
6	0.9631	N	0.9995	Y	1	Y
7	0.9631	N	1	Y	0.9998	Y
8	0.9992	Y	0.9628	N	1	Y
9	0.9617	N	0.9982	Y	1	Y
10	0.9996	Y	1	Y	0.9998	Y

**Table 3:** The number of feature parameters after dimensionality reduction.

	PCA	MDS	NGA	INGA
BP	17 ± 2	27 ± 3	21 ± 3	15 ± 2
SVM	2 ± 10	26 ± 4	20 ± 4	16 ± 3
RF	21 ± 4	22 ± 3	23 ± 3	10 ± 2
